# Study on the average speed of particles from a particle swarm derived from a stationary particle swarm

**DOI:** 10.1038/s41598-021-92402-w

**Published:** 2021-06-24

**Authors:** Tao Guo

**Affiliations:** Center for Drug Delivery System, Shanghai Institute of Materia Medica, Chinese Academy of Sciences, 501 Haike Road, Shanghai, 201210 China

**Keywords:** Fluid dynamics, Statistics

## Abstract

It has been more than 100 years since the advent of special relativity, but the reasons behind the related phenomena are still unknown. This article aims to inspire people to think about such problems. With the help of Mathematica software, I have proven the following problem by means of statistics: In 3-dimensional Euclidean space, for point particles whose speeds are *c* and whose directions are uniformly distributed in space (assuming these particles’ reference system is $\mathcal {R}_{0}$, if their average velocity is 0), when some particles (assuming their reference system is $\mathcal {R}_{u}$), as a particle swarm, move in a certain direction with a group speed *u* (i.e., the norm of the average velocity) relative to $\mathcal {R}_{0}$, their (or the sub-particle swarm’s) average speed relative to $\mathcal {R}_{u}$ is slower than that of particles (or the same scale sub-particle swarm) in $\mathcal {R}_{0}$ relative to $\mathcal {R}_{0}$. The degree of slowing depends on the speed *u* of $\mathcal {R}_{u}$ and accords with the quantitative relationship described by the Lorentz factor $\frac{c}{\sqrt{c^2-u^2}}$. Base on this conclusion, I have deduced the speed distribution of particles in $\mathcal {R}_{u}$ when observing from $\mathcal {R}_{0}$.

## Introduction

The research object of this article is the case in which the random motion (random motion in the following refers to motion with the speed of *c* and direction uniformly distributed in the Euclidean 3-dimensional space) of infinitely many point particles in infinite Euclidean space. It is difficult for us to track the trajectory of a point particle among a large number of randomly moving particles. In most cases, it is not necessary to obtain the trajectory of a single particle. All the information we perceive is usually the statistical average of a large number of particles. Therefore, statistical methods are very effective in solving this kind of problem.

As a symbolic mathematical calculation tool, Mathematica (*Wolfram Research Inc*.) can provide strong support for mathematical calculation and scientific exploration. Especially when the experimental scheme has been designed well and the specific mathematical calculations are more complex, the use of Mathematica can greatly improve efficiency. Version 8.0 (2010) of Mathematica introduced the symbolic statistics module for the first time and version 12.0 strengthened this module; the corresponding functions were very perfect, and some more difficult symbolic statistical problems were easily solved.

The random motion of particles in 3-dimensional Euclidean space has been historically studied in detail. Boltzmann^1^ studied the average speed of random collided gas molecules at a certain temperature and proposed the Maxwell distribution for the first time. On this basis, Maxwell^2^ developed the Maxwell distribution using a more rigorous approach. In this article, based on the Maxwell distribution, I use modern tools to study the relationship between the average velocity of randomly moving particles in different reference systems.

## Methods

Mathematica 12.1 for Mac (*Wolfram Research Inc*.) was used for all of the mathematical calculations, and the hardware was MacBook Pro (MD101CH/A) with an operating system of macOS High Sierra 10.13.6.

## Results and discussions

Suppose that the speeds of these particles (throughout this article, the “point particles” described in the above are called “particles”, “1st-order particles”, while larger finite-mass-level particles composed of *k* particles are called “*k*th-order particles”) are exactly the same (or $\sigma _1\ll c$, where *c* is the mean value of the particle speeds and $\sigma _1$ is their standard deviation), and the directions of their motions in 3-dimensional space are random. Therefore, these particles can be represented by random vectors with equal norms in Euclidean space. When a group of particles in the same 3-dimensional space is moving in one direction on average (i.e., their centroid is moving in one direction), they will lose some freedom of motion in other directions due to statistical effects, i.e., the movement trends in other directions will decrease. This phenomenon will be quantitatively explained in detail below.

Note that the velocity of a *k*th-order particle is the velocity of the overall center of mass of the *k* particles, which is the average of the velocity vectors of all these particles. Moreover, the projection of the velocity vector of a *k*th-order particle onto one of the three equivalent coordinate axes of the 3-dimensional Cartesian coordinate system is the mean value of the projection (onto the same axis) of the velocity vectors of the 1st-order particles forming the *k*th-order particle, which follow the same distribution; therefore, it approximately follows a normal distribution (central limit theorem). There are three equivalent (approximate) normal distributions, one on each of the three axes, which are not completely independent. However, Boltzmann^1^ and Maxwell^2^ proved that these distribution can, in fact, be equivalently treated as completely independent. This is because randomly selecting a vector is equivalent to randomly determining a three-axis coordinate; moreover, the problem of the momentum transfer of gas molecules participating in random collisions is also equivalent to the problem discussed in this article. Accordingly, the speeds of *k*th-order particles follow the Maxwell distribution. Suppose that the standard deviation of the projection (treated as a random variable; the same is done below) of the velocity of any one of the *k* equivalent particles forming a *k*th-order particle onto each equivalent coordinate axis is $\frac{\sigma }{\sqrt{k}}$. Then, the standard deviation of the projection of the velocity of a *k*th-order particle onto each equivalent coordinate axis is $\sigma$, namely the projection onto each coordinate axis follows a normal distribution with a mean value of 0 and a standard deviation of $\frac{\sigma }{\sqrt{k}}$. As a result, the speed of *k*th-order particles follows the Maxwell distribution with scale parameter $\frac{\sigma }{\sqrt{k}}$ (see Part 1 of the Supplementary Information for details).

As already mentioned, it is assumed that the speed of all particles is *c* (*c* > 0) and that the directions of their movement are evenly distributed in 3-dimensional space. Among the possible systems composed of randomly moving particles, the system with an average velocity of 0 is called the stationary reference system (denoted by $\mathcal {R}_{0}$), and a 3-dimensional Cartesian (rectangular) coordinate system *Oxyz* is established for it. A particle swarm formed by a subset of particles in a certain period of time and moving at an average velocity *u* is called a moving reference system (denoted by $\mathcal {R}_{u}$). Let the direction of the velocity of $\mathcal {R}_{u}$ be parallel to the *z*-axis in the direction of increasing *z*. Then, the mean value of the velocity component of the particles in $\mathcal {R}_{u}$ along the *z*-axis must be *u*. Under the assumptions that all particles in $\mathcal {R}_{u}$ are represented by vectors with their starting points at the origin of the coordinate system and that the point (0, 0, *u*) is taken as the dividing point of the *z*-axis, the vectors in $\mathcal {R}_{u}$ can be separated into two groups: the components of the vectors above this dividing point and the components of the vectors below it. These vectors randomly enter $\mathcal {R}_{u}$ from $\mathcal {R}$ $_{0}$ with equal probability. Therefore, the distribution of the vectors in $\mathcal {R}_{u}$ can be thought of as a mixed distribution of the vector distribution of the components above the dividing point and the vector distribution of the components below the dividing point. When the mean value of the components on the *z*-axis of this mixed distribution is *u*, the mixture weights *w* can be determined. With this value as the reference, the distribution of the vectors that form the mixed distribution on the *x*-axis (or *y*-axis) can be determined; thus, their standard deviation $\sigma _{u}$ can also be obtained. When the standard deviation of the components on the *z*-axis of this mixed distribution is also $\sigma _{u}$, then the speed of *k*th-order particles (of mass $\mu k$, where $\mu$ is the mass of a single particle; the same is true below) in $\mathcal {R}_{u}$ follows the Maxwell distribution with scale parameter $\sigma _{u,k}$, where

1 $$\begin{aligned} \sigma _{u,k}=\frac{\sigma _{u}}{\sqrt{k}}. \end{aligned}$$

Therefore, $\sigma _{u,k}$ is directly proportional to the average speed $\overline{v}_{u,k}$ of the *k*th-order particle, namely,

2 $$\begin{aligned} \overline{v}_{u,k}=2\sqrt{\frac{2}{\pi }}\sigma _{u,k}. \end{aligned}$$

By substituting Eq. (1) into Eq. (2), we obtain

3 $$\begin{aligned} \overline{v}_{u,k}=2\sqrt{\frac{2}{\pi }}\cdot \frac{\sigma _{u}}{\sqrt{k}}. \end{aligned}$$

The distribution of the vectors in $\mathcal {R}_{0}$ is relatively simple. Suppose that the standard deviation of their components on the *x*-axis (or *y*- or *z*-axis) is $\sigma _{0}$; similarly, the average velocity of the *k*th-order particles that is formed by them is

4 $$\begin{aligned} \overline{v}_{0,k}=2\sqrt{\frac{2}{\pi }}\cdot \frac{\sigma _{0}}{\sqrt{k}}. \end{aligned}$$

When particles of the same mass level are formed in both $\mathcal {R}_{u}$ and $\mathcal {R}_{0}$, the ratio between their average speeds (Eq. 3 to Eq. 4) is

5 $$\begin{aligned} \frac{\overline{v}_{u,k}}{\overline{v}_{0,k}}=\frac{\sigma _{u}}{\sigma _{0}}. \end{aligned}$$

Therefore, the ratio of $\sigma _{u}$ to $\sigma _{0}$ is the ratio between the average speeds of particles of higher mass levels in $\mathcal {R}_{u}$ and $\mathcal {R}_{0}$. A more detailed introduction will be presented in the following.

As mentioned above, in the 3-dimensional Cartesian coordinate system constructed in the stationary reference system $\mathcal {R}_{0}$, if the moving reference system $\mathcal {R}_{u}$ moves along the *z*-axis at velocity *u*, then the *x*- and *y*-coordinates are equivalent; hence, only the *x*-coordinate is considered in the following. In view of the nature of probability theory, in $\mathcal {R}_{0}$, if the components of these vectors along the *z*-axis are uniformly distributed in the interval [−*c*, *c*], then the probability density on the *x*-axis is

6 $$\begin{aligned} \mathcal {D}(\theta ,\eta )=c\cdot \cos \theta \cdot \sin \cos ^{-1}\eta , \end{aligned}$$

where the random variables are $\Theta \sim U(-\pi , \pi )$ and $H \sim U(-1, 1)$. Note that in this article, random variables (vectors) are expressed in capital letters, and the values of random variable (vectors) are expressed in the corresponding lower-case letters. The component distribution of the vectors whose components are above (0, 0, *u*) on the *x*-axis is denoted by $\mathcal {D}_1$, and its probability density is written as

7 $$\begin{aligned} \mathcal {D}_1(\theta ,\eta )=c\cdot \cos \theta \cdot \sin \cos ^{-1}\eta , \end{aligned}$$

where the random variables are $\Theta \sim U(-\pi , \pi )$ and $H \sim U(\frac{u}{c}, 1)$. Correspondingly, the component distribution of these vectors on the *z*-axis is denoted by $\mathcal {D}_3$, namely, $\mathcal {D}_3\sim U(u, c)$. The component distribution of the vectors whose components are below (0, 0, *u*) on the *x*-axis is denoted by $\mathcal {D}_2$, and its probability density is written as

8 $$\begin{aligned} \mathcal {D}_2(\theta ,\eta )=c\cdot \cos \theta \cdot \sin \cos ^{-1}\eta , \end{aligned}$$

where the random variables are $\Theta \sim U(-\pi , \pi )$ and $H \sim U(-1, \frac{u}{c})$. Correspondingly, the component distribution of these vectors on the *z*-axis is denoted by $\mathcal {D}_4$, namely, $\mathcal {D}_4\sim U(-c, u)$. When the mean value of the components of the mixed distribution consisting of $\mathcal {D}_3$ and $\mathcal {D}_4$ on the *z*-axis is *u*, the corresponding mixture weights are $\frac{c+u}{2c}$ and $\frac{c-u}{2c}$, respectively. Note that $\mathcal {D}_1$ and $\mathcal {D}_2$ are randomly selected from the vector swarms with the same characteristics as $\mathcal {D}_3$ and $\mathcal {D}_4$, respectively. Then, the mixed distribution consisting of $\mathcal {D}_1$ and $\mathcal {D}_2$ can be calculated in accordance with these two weights (the analytical form of this mixed distribution cannot be given in this article at present); then, it can be found that the standard deviation of the velocity components on the *x*-axis of the particles in $\mathcal {R}_u$ is

9 $$\begin{aligned} \sigma _{u}=\frac{\sqrt{c^2-u^2}}{\sqrt{3}}. \end{aligned}$$

By evaluating the ratio between Eq. (9) and the standard deviation of the velocity components on the *x*-axis of the particles in $\mathcal {R}_0$, we can obtain the corresponding scale factor, namely,

10 $$\begin{aligned} \frac{\sqrt{c^2-u^2}}{c}. \end{aligned}$$

This is equivalent to the additive inverse of the Lorentz factor when *c* represents the speed of light. Obviously, the ratio of the standard deviations of the velocity components on the *y*-axis is also this scale factor, as shown in Eq. (10). This same factor can also be obtained by evaluating the ratio of the standard deviation of the velocity components on the *z*-axis of the mixed distribution in $\mathcal {R}_u$ to the standard deviation of the velocity components on the *z*-axis in $\mathcal {R}_0$. The detailed Mathematica code for the above calculation can be found in Part 2 of the Supplementary Information.

Based on the above conclusions, the following result will be easily obtained: The abovementioned case is the movement of the particle swarm relative to $\mathcal {R}_u$ observed from $\mathcal {R}_0$. If the movement of the particle swarm in $\mathcal {R}_u$ relative to $\mathcal {R}_0$ are observed from $\mathcal {R}_0$, the probability density of the magnitude of the momentum of the particle swarm formed by *k* particles in $\mathcal {R}_u$ relative to $\mathcal {R}_0$ observed from $\mathcal {R}_0$ can be obtained based on the above conclusions, namely,

11 $$\begin{aligned} \frac{\sqrt{3} x \left( \mathrm {e}^{\frac{6 u x}{c^2-u^2}}-1\right) \mathrm {e}^{-\frac{3 (k u+x)^2}{2 k \left( c^2-u^2\right) }}}{k u \sqrt{2 \pi k \left( c^2-u^2\right) }}. \end{aligned}$$

The detailed Mathematica code for the above calculation can be found in Part 3 of the Supplementary Information.

## Conclusions

This result implies that when a subset of the particles in the reference system $\mathcal {R}_0$ composed of particles moving at the same speed (such as *c*) and in (spatial) random directions forms a reference system $\mathcal {R}_u$ moving at speed *u*, the speed of the particles or their forming *k*th-order ($k\in \mathbb {N}$ and $k\ne 1$) particles in $\mathcal {R}_u$ will be relatively decreased, with a degree of deceleration corresponding to the value determined by the scale factor given by Eq. (10). Base on this conclusion, the speed distribution of particles in $\mathcal {R}_u$ when observing from $\mathcal {R}_0$ have also been solved.

It is also noted that in $\mathcal {R}_u$, the slowdown on all three axes is the same. This means that there is no difference in physical laws that can be perceived between $\mathcal {R}_u$ and the stationary reference system $\mathcal {R}_0$. Therefore, when another moving reference system $\mathcal {R}_{u'}$ appears in $\mathcal {R}_u$, $\mathcal {R}_u$ can, in turn, be treated as a stationary reference system, which is a useful feature. This reveals that any reference system that satisfies the conditions given in the physical model can be regarded as a stationary reference system, regardless of whether it is an absolutely stationary reference system. At the same time, any $\mathcal {R}_u$ randomly generated in $\mathcal {R}_0$ (when *u* is fixed) is equivalent to $\mathcal {R}_u$ which can be regarded as a stationary reference system, and is the same $\mathcal {R}_u$ without physical law difference. Therefore, any $\mathcal {R}_u$ (as an undifferentiated particle set) can be regarded as generated randomly by $\mathcal {R}_0$ with relative motion (the speed is *u*) to $\mathcal {R}_u$. In this way, $\mathcal {R}_0$ (as an undifferentiated particle set) can also be regarded as randomly generated by $\mathcal {R}_u$ with relative motion (the speed is *u*) to $\mathcal {R}_0$. The principle of the special relativity effect of moving particles in space is the statistical effect of randomly moving particles.

## Supplementary information

Supplementary Information.
