# Supplementary material for: Study on the average speed of particles from a particle swarm derived from a stationary particle swarm
Source: Sci Rep. 2021 Jun 24;11:13290. doi: 10.1038/s41598-021-92402-w (PMC8225804; doi:10.1038/s41598-021-92402-w)

# Supplementary Information
(Mathematica v12.1 code of TraditionalForm)

## Part 1. The Ratio of the Standard Deviations Equals the Ratio of the Average Speeds for the Same Mass Level Particles in Different References

Definition: Particles with a higher mass level composed of $k$ particles are called $k$th-order particles. Then, the velocity of a $k$th-order particle is the velocity of the overall center of mass of the $k$ particles, which is the average of the velocity vectors of all these particles.
Assumption: Each particle is moving at the same speed and in a random direction in space.
Thus, the projection of the velocity vector of a $k$th-order particle onto one of the three equivalent coordinate axes of the 3-dimensional Cartesian coordinate system is the mean value of the projection (onto the same axis) of the velocity vectors of the 1st-order particles forming the $k$th-order particle, which follow the same distribution; therefore, it approximately follows a normal distribution (central limit theorem).
There are three equivalent (approximate) normal distributions, one on each of the three axes, which are not completely independent. However, James Clerk Maxwell and Ludwig Boltzmann proved that these distribution can, in fact, be equivalently treated as completely independent. This is because randomly selecting a vector is equivalent to randomly determining a three-axis coordinate; moreover, the problem of the momentum transfer of gas molecules participating in random collisions is also equivalent to the problem discussed in this article.

First, the probability density of the norm of the 3-dimensional vectors formed by three normal distribution $N(0, \sigma_2)$ components that are independent on three coordinate axes is calculated.

```
Clear["Global`*"];
𝒟 = Simplify[PDF[TransformedDistribution[x^2 + y^2 + z^2,
        {x, y, z} ≈ ProductDistribution[{NormalDistribution[0, σ2], 3}]], x], Assumptions → σ2 > 0];
𝒟1 = PDF[TransformedDistribution[ √x , x ≈ ProbabilityDistribution[𝒟, {x, 0, +∞}]], x]
```

Out[•]=
$$\begin{cases} \frac{\sqrt{\frac{2}{\pi}}\, x^2\, e^{-\frac{x^2}{2\sigma_2^2}}}{\sigma_2^3} & x > 0 \\ 0 & \text{True} \end{cases}$$

Then, we find the probability density of the Maxwell distribution with scale parameter $\sigma_2$:

In[•]:=
```
𝒟2 = PDF[MaxwellDistribution[σ2], x]
```

Out[•]=
$$\begin{cases} \frac{\sqrt{\frac{2}{\pi}}\, x^2\, e^{-\frac{x^2}{2\sigma_2^2}}}{\sigma_2^3} & x > 0 \\ 0 & \text{True} \end{cases}$$

Therefore, these two probability densities are equal:

In[•]:=
```
𝒟1 − 𝒟2
```

Out[•]= 0

We verify the above conclusion ($c$ is the speed of the 1st-order; $n$ is the number of vectors) (This code takes approximately 166 seconds):

```
In[∘]:= c = 1;
n = 1000;
m = 1 000 000;
dd = {};
ProgressIndicator[Dynamic[i], {1, m}]
For[i = 1, i < m, i++,
    𝓗 = RandomPoint[Sphere[{0, 0, 0}, c], n];
    𝓗𝓗 = Norm[Total /@ Transpose[𝓗]];
    dd = AppendTo[dd, 𝓗𝓗]];
𝒟 = SmoothKernelDistribution[dd, {"Adaptive", Automatic, Automatic}];
Plot[{PDF[𝒟, x], PDF[MaxwellDistribution[c/√3 √n], x]},
  {x, 0, 100 c}, PlotStyle → {{Red, Thickness → 0.0032}, {Blue, Thickness → 0.0032}},
  Frame → {{True, False}, {True, False}}, FrameStyle → Directive[Black, Thickness → 0.0017],
  LabelStyle → Directive[Black, FontFamily → "Arial", FontSize → 14],
  Epilog → Inset[LineLegend[{Directive[Blue, Thickness[0.0032]], Directive[Red, Thickness[0.0032]]},
      {Style["Theoretical", FontFamily → "Arial", FontSize → 14],
        Style["Simulated", FontFamily → "Arial", FontSize → 14]}, LegendFunction →
        (Framed[#, RoundingRadius → 4, FrameStyle → GrayLevel[.6]] &)], Scaled[{0.732, 0.644}]]]

Out[∘]=

Out[∘]=
```

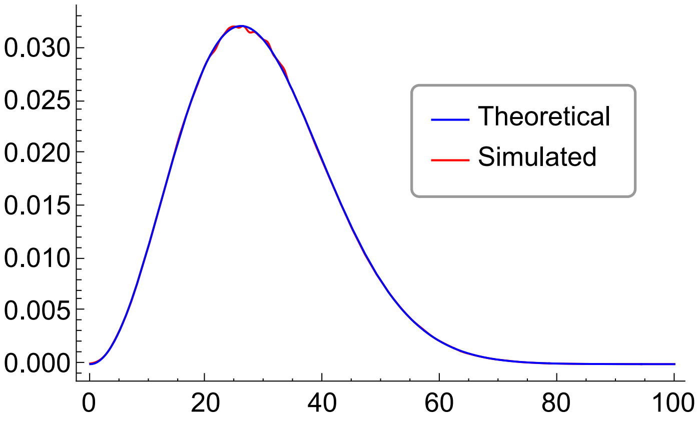


Accordingly, the norm of the 3-dimensional vectors formed by three normal distribution $N(0, \sigma_2)$ components which are independent on three coordinate axes follows the Maxwell distribution with the scale parameter $\sigma_2$.

Suppose that the standard deviation of the projection of the velocity of any one of the $k$ equivalent particles forming a $k$th-order particle onto each equivalent coordinate axis is $\sigma$. Then, the standard deviation of the projection of the velocity of a $k$th-order particle onto each equivalent coordinate axis (i.e., the mean value of the projection of the velocity of 1st-order particle) is $\frac{\sigma}{\sqrt{k}}$, namely, the projection onto each coordinate axis (approximate) follows a normal distribution with a mean value of 0 and a standard deviation of $\frac{\sigma}{\sqrt{k}}$. As a result, the speed of $k$th-order particles follows the Maxwell distribution with scale parameter $\frac{\sigma}{\sqrt{k}}$.

Then, the average velocity of the $k$th-order particles is

In[•]:= $\bar{v}$ = Mean[MaxwellDistribution[$\frac{\sigma}{\sqrt{k}}$]]

Out[•]= $\frac{2\sqrt{\frac{2}{\pi}}\,\sigma}{\sqrt{k}}$

For the $k$th-order particles in different references ($\mathcal{R}_u$ and $\mathcal{R}_0$) and with different standard deviations ($\sigma_u$ and $\sigma_0$), the ratio of their average velocity $\bar{v}_u / \bar{v}_0$ =

In[•]:= $\frac{2\sqrt{\frac{2}{\pi}}\,\sigma_u}{\sqrt{k}} \Big/ \frac{2\sqrt{\frac{2}{\pi}}\,\sigma_0}{\sqrt{k}}$

Out[•]= $\frac{\sigma_u}{\sigma_0}$

Therefore, the ratio of $\sigma_u$ to $\sigma_0$ is the ratio between the average speeds of particles of higher mass levels in $\mathcal{R}_u$ and $\mathcal{R}_0$.

## Part 2. The Process of Obtaining the Lorentz Factor for Randomly Moving Particles

Correspondence:

The mixed distribution of $\mathcal{D}_1$and $\mathcal{D}_2$ is represented by $\mathcal{D}$12;

The mixed distribution of $\mathcal{D}_3$and $\mathcal{D}_4$ is represented by $\mathcal{D}$34;

The rest of the symbols are consistent with those in the main text.

```
Clear["Global`*"];
𝒟 = TransformedDistribution[c Cos[θ] Sin[ArcCos[η]],
    {θ ≈ UniformDistribution[{−π, π}], η ≈ UniformDistribution[{−1, 1}]}];
𝒟1 = TransformedDistribution[c Cos[θ] Sin[ArcCos[η]],
    {θ ≈ UniformDistribution[{−π, π}], η ≈ UniformDistribution[{u/c, 1}]}];
𝒟2 = TransformedDistribution[c Cos[θ] Sin[ArcCos[η]],
    {θ ≈ UniformDistribution[{−π, π}], η ≈ UniformDistribution[{−1, u/c}]}];
𝒟3 = TruncatedDistribution[{u, c}, UniformDistribution[{−c, c}]];
𝒟4 = TruncatedDistribution[{−c, u}, UniformDistribution[{−c, c}]];
𝒟34 = MixtureDistribution[{w, 1 − w}, {𝒟3, 𝒟4}];
Simplify[Mean[𝒟34], Assumptions → 0 < u < c]
```

Out[•]= $\frac{1}{2}(c\,(2\,w-1)+u)$

Let the mean value expression be $\frac{1}{2}(c\,(2\,w-1)+u) = u$, then find the weight $w$

In[•]:= Reduce[$\frac{1}{2}$ (c (2 w − 1) + u) == u, w]

Out[•]= $(u = 0 \wedge c = 0) \vee \left(c \neq 0 \wedge w = \frac{c+u}{2\,c}\right)$

Then, the mixed distribution $\mathcal{D}12$ consisting of $\mathcal{D}_1$ and $\mathcal{D}_2$ can be calculated in accordance with this weight $w$. The analytical form of $\mathcal{D}12$ cannot be given by Mathematica. Therefore, the standard deviation of $\mathcal{D}12$ is calculated directly.

In[∘]:= $w = \frac{c+u}{2c}$;
$\mathcal{D}12$ = MixtureDistribution[{$w$, 1 − $w$}, {$\mathcal{D}_1$, $\mathcal{D}_2$}];
$\sigma_u$ = Simplify[StandardDeviation[$\mathcal{D}12$], Assumptions → 0 < $u$ < $c$]

Out[∘]= $\frac{\sqrt{c^2 - u^2}}{\sqrt{3}}$

The standard deviation of $\mathcal{D}34$ is the same.

In[∘]:= Simplify[StandardDeviation[$\mathcal{D}34$], Assumptions → 0 < $u$ < $c$]

Out[∘]= $\frac{\sqrt{c^2 - u^2}}{\sqrt{3}}$

Then, the ratio between $\sigma_u$ and the velocity components on the $x$-axis of the particles in $\mathcal{R}_0$ can be obtained.

In[∘]:= Simplify[$\sigma_u$ / StandardDeviation[$\mathcal{D}$], Assumptions → 0 < $u$ < $c$]

Out[∘]= $\frac{\sqrt{c^2 - u^2}}{c}$

The same factor can also be obtained by evaluating the ratio of the standard deviation of $\mathcal{D}34$ to the standard deviation of the velocity components on the $z$-axis in $\mathcal{R}_0$.

In[∘]:= Simplify[StandardDeviation[$\mathcal{D}34$] / StandardDeviation[UniformDistribution[{−$c$, $c$}]],
Assumptions → 0 < $u$ < $c$]

Out[∘]= $\frac{\sqrt{c^2 - u^2}}{c}$

## Part 3. The Probability Density of the Magnitude of the Momentum of the Particle Swarm in $\mathcal{R}_u$ Relative to $\mathcal{R}_0$ Observed from $\mathcal{R}_0$

Based on the above conclusions, the following result will be easily obtained:

When observing all of the moving particles in $\mathcal{R}_u$ from $\mathcal{R}_0$, all the randomly moving particles in $\mathcal{R}_u$ can be considered to have an additional velocity component $u$ along the $z$-axis. Then, according to cosine theorem, the probability density of the particles in $\mathcal{R}_u$ observed in $\mathcal{R}_0$ can be expressed as (where $k$ is the number of the particles, $u$ is the speed of $\mathcal{R}_u$ and $v$ is the norm of momentum of these $k$ particles observed from $\mathcal{R}_u$):

In[•]:=
```
Clear["Global`*"];
𝒟 = TransformedDistribution[Sqrt[(k u)^2 + v^2 - 2 k u v Cos[ArcCos[η]]],
    {v ≈ MaxwellDistribution[(Sqrt[k] Sqrt[c^2 - u^2])/Sqrt[3]], η ≈ UniformDistribution[{-1, 1}]}];
FullSimplify[PDF[𝒟, x], Assumptions → c > 0 ∧ 0 < u < c]
```

Out[•]=

$$\begin{cases} \dfrac{\sqrt{3}\, x \left(e^{\frac{6 u x}{c^2-u^2}}-1\right) e^{-\frac{3 (k u+x)^2}{2 k \left(c^2-u^2\right)}}}{k u \sqrt{2 \pi c^2 k-2 \pi k u^2}} & k>0 \land ((x>0 \land k u>x) \lor k u<x) \\ -\dfrac{\sqrt{6 \pi} \sqrt{c^2 k-u x} \left(5 u x-2 c^2 k\right) \operatorname{erf}\left(\frac{\sqrt{6}\, x}{\sqrt{c^2 k-u x}}\right)+4 x e^{\frac{6 x^2}{u x-c^2 k}} \left(c^2 (6 k+2)-u (2 u+3 x)\right)-8 x (c-u) (c+u)}{4 \sqrt{6 \pi}\, k^{5/2} u ((c-u) (c+u))^{3/2}} & k u=x \land k>0 \end{cases}$$

The meaningful part (first branch) is selected to be verified. Note that the sampling with the replacement method in the particle swarm with a mean speed of $u$ can simulate all of the cases of the particle swarm with a mean speed of $u$. (The following code takes averagely 108 + 77 minutes)

In[∘]:=
```
c = 1;
n = 1 000 000;
ℋℋ = 0;
While[ℋℋ < 2700,
    ℋ = RandomPoint[Sphere[{0, 0, 0}, c], n];
    ℋℋ = Norm[Total /@ Transpose[ℋ]]];
m = 100 000;
dd = {};
ProgressIndicator[Dynamic[j], {1, m}]
For[j = 1, j < m, j++,
    ℋ0 = RandomChoice[ℋ, 0.3 n];
    ℋℋ0 = Norm[Total /@ Transpose[ℋ0]];
    dd = AppendTo[dd, ℋℋ0]];
𝒟 = SmoothKernelDistribution[dd, {"Adaptive", Automatic, Automatic}];
k = 0.3 n;
```

$$u = \frac{\mathcal{HH}}{n};$$

$$\text{Plot}\left[\left\{\text{PDF}[\mathcal{D}, x], \frac{\sqrt{3}\, x \left(e^{\frac{6 u x}{c^2-u^2}} - 1\right) e^{-\frac{3 (k u + x)^2}{2 k (c^2-u^2)}}}{k\, u\, \sqrt{2 \pi c^2 k - 2 \pi k u^2}}\right\}, \{x, 0, 2500\},\right.$$

```
  PlotStyle → {{Red, Thickness → 0.0032}, {Blue, Thickness → 0.0032}},
  Frame → {{True, False}, {True, False}}, FrameStyle → Directive[Black, Thickness → 0.0017],
  LabelStyle → Directive[Black, FontFamily → "Arial", FontSize → 14],
  Epilog → Inset[LineLegend[{Directive[Blue, Thickness[0.0032]], Directive[Red, Thickness[0.0032]]},
        {Style["Theoretical", FontFamily → "Arial", FontSize → 14],
          Style["Simulated", FontFamily → "Arial", FontSize → 14]}, LegendFunction →
          (Framed[#, RoundingRadius → 4, FrameStyle → GrayLevel[.6]] &)], Scaled[{0.753, 0.644}]]]
```

Out[∘]=

Out[∘]=

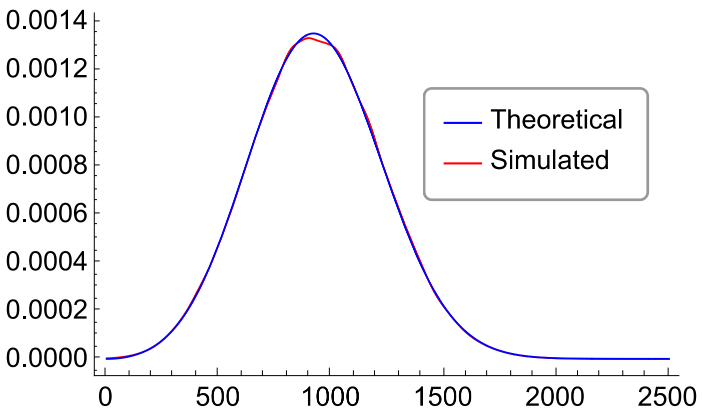

Supplement: Supplementary file 1 — Supplementary Information. [file 41598_2021_92402_MOESM1_ESM.pdf]
